# American Dermatological Association

**Published:** 1881-12

**Authors:** 


### ARTICLE VIII.

AMERICAN DERMATOLOGICAL ASSOCIATION: FIFTH ANNUAL MEETING. Held at Newport, R. I., August 30 and 31, and Sept. 1, 1881. Official Report of the Proceedings by the Secretary, Dr. ARTHUR VAN HARLINGEN, of PHILADELPHIA.

[CONCLUDED FROM NOVEMBER NUMBER.]

### SECOND DAY.—MORNING SESSION.

DR. ISAAC E. ATKINSON, of Baltimore, read a paper entitled

**A Case of Tubercular Leprosy,**

which was supplemented by a paper by Dr. I. Bermann, of Baltimore, upon the presence of the "bacillus lepræ" in the leprous infiltrations removed from the patient. The patient, a woman about 45 years old, was born of healthy parents, in Baltimore, and had never been beyond the limits of Maryland. She was married at sixteen. Her illness dated back about five years, and had followed a course typical of tubercular leprosy. The most interesting feature of the case was the probability of its having been acquired through contagion. This is the only case upon record of leprosy acquired by one who, born in Maryland, has never been beyond its limits. It can hardly, therefore, be an accidental coincidence that this woman, living for some time next door to a leper, whose case has been recorded by Dr. Rohé in the *Maryland Medical Journal* (1878), should get the disease without deriving it from him. It should be remembered that this man, who probably acquired his leprosy in Cuba, a young man, a native of Barbadoes, and a woman who probably acquired her disease in Louisiana (all reported by Rohé), comprise all the lepers that have been reported in Maryland for a number of years. It is evident that leprosy is one of the rarest of maladies in that State. The objection to evidence adduced in support of the contagiousness of leprosy has heretofore been that it has been collected in countries or localities where the disease prevails as an epidemic, and might arise from other causes. Here, however, is a case where the contagious influence was possible in a locality where leprosy not only does not prevail, but has never, previously, been known to affect one whose life has been spent within its limits. Such conditions have never before been reported. The researches of Dr. Bermann into the histological pathology of the tubercles removed from the ear of this patient, enabled him to confirm the discoveries of Hauser, Neisser, Eklund, and others, of a bacterium as a constant attendant and probable cause of leprosy. Aniline colors being best for demonstrating bacteria, Bermann followed the prescribed methods and detected in the nuclei of the cells little bodies corresponding exactly with the bacillus lepræ. The process giving the most perfect results was that of staining the section in a one per cent. solution of aniline blue for a few seconds, washing in absolute alcohol several times, transferring to oil of cloves, again to the alcohol, then back to the oil of cloves, and finally mounting in balsam. The result of this method is, that the pigment is nearly removed from the protoplasm of the cell and leaves the bacillus deeply stained. Several prepared sections were exhibited.

On motion, it was agreed to postpone the discussion on Dr. Atkinson's paper until the other papers on the same subject should have been read.

DR. J. NEVINS HYDE, of Chicago, read a paper entitled

**Study of a Case of Acute Tubercular Leprosy.**

This paper was illustrated by a portrait in oil, representing the face of the patient.

DR. HYDE then read a paper by DR. H. D. SCHMIDT, of New Orleans, entitled

**The Pathology of Leprosy.***

DR. LOUIS A. DUHRING, of Philadelphia, was announced to read a paper by MR. WM. FLETCHER, B.A., of Canada, entitled

**Cases of Leprosy in Cape Breton, Nova Scotia.**

Dr. Duhring apologized for the non-reception of Mr. Fletcher's communication, which had miscarried in the mail.

On motion of DR. WHITE, of Boston, it was agreed that Mr. Fletcher's paper be referred to Dr. Graham, of Toronto, the member of the Committee on Statistics in whose district the

* This paper will appear in the *Archives of Medicine*, October No.

cases had occurred, in order that it might be incorporated with the report of the committee.

### SECOND DAY.—EVENING SESSION.

### Discussion on Leprosy.

DR. WHITE, of Boston, said he was becoming more and more convinced of the contagiousness of leprosy through inoculation. The rise and spread of the disease in the Sandwich Islands seemed convincing as to contagiousness. He asked Dr. Atkinson if he could explain how contagion had occurred in his case.

DR. ATKINSON, of Baltimore, in reply to Dr. White, said that there were open sores upon the man B., which of course might be the means of contagion. Whether sexual intercourse had taken place between the parties he could not say, but every outward circumstance was against such a supposition. The woman was a person of the highest respectability.

DR. WHITE went on to say, regarding Dr. Hyde's paper, that he could not accept the case there reported as one of leprosy. The rapidity of development of the skin lesions was unprecedented. The picture accompanying the paper did not seem to him to present the appearance of leprosy. He would be more ready to accept the diagnosis after a careful microscopical examination of the lesions should have been made.

DR. GRAHAM, of Toronto, said that Dr. Keys, who had examined the lepers at Cape Breton, considered leprosy contagious only by inoculation. Drs. Bayard and Wilson were of the opinion that the disease is contagious.

DR. HEITZMANN, of New York, said, in reference to the microscopic preparations of leprosy made by Dr. Bermann and exhibited by Dr. Atkinson, that these specimens showed appearances which some observers considered parasites. The brief space of time available for the examination, and the method of mounting employed, prevented him from expressing a positive opinion, but he considered the supposed rods to be a part of the protoplasmic structure of the so-called cell. He thought staining agents delusive. As to Dr. Schmidt's elaborate paper, although this was valuable, it was based upon old-fashioned views of pathology. As we know that there are trophic centers everywhere in the central nervous system, and as many skin diseases appear in the light of recent investigations to be due to affections of the central nervous system, it was to this system, in his opinion, we must look for the origin of leprosy.

DR. DUHRING, of Philadelphia, said he had been much interested in hearing Dr. Atkinson's paper. In listening to the history of the case as given, he could see no point at which contagion could have taken place. The opinion of observers seems more and more inclining to the view of the contagiousness of leprosy. He agreed with Dr. White in thinking that contagion could only take place by inoculation. With regard to the case which had been reported by the President, Dr. Hyde, Dr. Duhring inclined to the belief that contagion could only have taken place by inoculation. With regard to Dr. Hyde's case, so far as he could judge from what he had heard of the history, he thought the diagnosis doubtful. He had never heard of a case of leprosy running such a rapid course. From what he had heard of the case, and especially from the accompanying picture, he was reminded of a case described by himself in a paper read before the Association a few years ago, under the name of "Inflammatory Fungoid Neoplasm." The color and appearance of the lesions in Dr. Hyde's case resembled closely those observed in Gubler's, Piffard's, and his own case.

THE PRESIDENT, DR. HYDE, of Chicago, said that he himself was not perfectly certain of the diagnosis. He should not come to a positive conclusion until a careful histological examination had been made of the lesions. He had at first regarded the case as one of that affection called by Dr. Heitzmann "myeloma," and of which Dr. Duhring's case was an instance. As to the acuteness of the disease, it must be remembered that it had in fact lasted a long time. Dr. Hyde then recapitulated the chief points in his description of the case, going to show that the lesions were characteristic to a considerable degree of leprosy.

DR. ATKINSON, said that the fortuitous concurrence of two cases, such as had been described in his paper, would seem almost impossible. The only case of leprosy which had ever originated in the State of Maryland was the one which he had described, and this had occurred in the immediate neighborhood of a former case. He thought it impossible to escape the conclusion that there was some connection between the two cases, even though the history had failed to bring such connection to light. In his opinion, the hereditability of leprosy was doubtful. Regarding the question of the presence of "bacillus lepræ" in the microscopic specimens, he would only say that Dr. Bermann claims that he has found similar appearances to those described by Hansen and Ecklund, to which they gave that name.

DR. WIGGLESWORTH, of Boston, said that he had in his student days seen much leprosy, having traveled in Norway, Spain and Turkey for the purpose of studying the disease. He had, however, never seen a case of leprosy like the case described by Dr. Hyde. If Dr. Hyde could see Baretta's model of a case of "mycosis fungoide," he would see how closely it resembled his (Dr. Hyde's) case. Dr. Wigglesworth thought that many of these diseases might be grouped as lymphosarcoma, and went on to give the leading symptoms of Dr. Hyde's case and to point out the resemblance between it and lymphosarcoma.

DR. HYDE said he had been lead by reading Kaposi's paper on a case of acute leprosy to incline to this diagnosis in the case reported.

DR. DUHRING said he regarded Baretta's model, to which Dr. Wigglesworth had alluded, as representing the same disease as his inflammatory fungoid neoplasm. He referred to a case of leprosy which he had seen where the course was very obscure. It had occurred in a Cuban gentleman who had traveled much, and in whom it had shown itself at the age of thirty, the patient dying five years later.

DR. WIGGLESWORTH recalled the case of an American sailor who visited various ports of South America, and returning showed symptoms of leprosy seven years later. He thought the disease might have been contracted in this case by innoculation, by cohabitation, etc.

DR. EDWARD WIGGLESWORTH, of Boston, read a paper on

**Buccal Ulcerations of Constitutional Origin.**

These are usually syphilitic in nature, though they are not always so. The fact that syphilitic ulcerations sometimes occur in persons above all suspicion of sexual immorality makes it doubly important for the general practitioner to recognize them by their appearance without asking too many questions. The only maladies likely to occasion doubt in regard to diagnosis are lupus and tuberculosis, and occasionally epithelioma. In cases of lupus the local treatment is identical with that adapted to ulcerations due to syphilis. When tuberculosis exists with ulcerations of the fauces or palate, or both, the pulmonary symptoms are almost always far advanced, and even when they cannot be detected the patient is sure to die of tuberculosis, more or less general, and treatment is practically useless. The possibility, however, that the case may be one or either of these diseases should always make the practitioner careful in expressing an opinion and considerate in his language, for the peace of a whole family is often at stake.

The writer then went on to give notes of a series of illustrative cases, and followed these by some general remarks on the treatment, particularly of syphilitic ulcerations. Stimulants are better than caustics, and, indeed, much harm is done by the indiscriminate use of the latter. The parts must be kept well cleansed, and this alone, when the ulceration is in the pharynx, gives great relief. The writer recommended the following solution for atomization :

℞ Tinct. iodini, pts. v;
Glycerinæ, pts. x;
Aquæ, pts. xxx.—M.

The most convenient instrument to use is that known as Fulgraffe's, which is made of hard rubber, with nozzles of various shapes—one to send a stream of spray up behind the soft palate, one for a horizontal stream, and one for use in the larynx. The bottle should be kept nearly full, so that the stream may not be kept up longer than is intended, owing to compressed air remaining in the bottle. Three or four applications of twenty seconds each will be sufficient to thoroughly cleanse and stimulate any ordinary ulceration.

After the use of the spray, iodoform should be applied to the ulcer with a camel's hair pencil, or blown on to it by an insufflator. A little powdered gum arabic mixed with the iodoform causes it to adhere better. Iodoform can also be conveniently deposited on the nasal mucous membrane by letting the patient inhale a saturated solution of the drug in ether.

Some cases require the use of the nasal douche with disinfectants. A convenient formula is one teaspoonful of chlorate of potassium with one-third as much carbonate of sodium and fifteen drops of a five-per-cent. solution of permanganate of potassium, all in a quart of water at 25 degrees C., used with a fountain syringe. It is well to remember that a spray or a powder carried by a current of air may be used by blowing it into one nostril while the patient breathes through the open mouth. The spray then passes through the vault of the pharynx and escapes by the other nostril. If desirable, by instructing the patient to breathe through the nose, a powder may be blown into the larynx through the nostril.

Constitutional treatment is of course necessary. The lesions occurring late in the disease—from the second to the twentieth year, and usually in cachectic subjects—iodine must play a more important part than mercury. Cod-liver oil, ferric iodide, potassic iodide, iodine spray, and iodoform—these are the great remedies. Of course all hygienic measures must be united with these.

DR. F. B. GREENOUGH, of Boston, thought that although local treatment was beneficial in the class of cases described, yet keeping the parts clean, and constitutional (mixed) treatment was as good or better.

DR. WIGGLESWORTH said he thought a cure could be effected more rapidly by employing local treatment in addition to cleanliness and constitutional measures.

DR. GREENOUGH said he had found iodoform a very useful application. He had used it successfully in one case of epithelioma.

DR. HEITZMANN, of New York, asked Dr. Wigglesworth if he had not found much difficulty in distinguishing between syphilis and tuberculosis of the throat. He adduced several personal instances where the diagnosis was very difficult.

DR. WIGGLESWORTH said he often found the diagnosis between syphilitic and tubercular ulceration of the throat very difficult. In answer to a question by the President he said the form of iodide of iron he preferred was the syrupus ferri iodidi of the pharmacopœia.

DR. ATKINSON, of Baltimore, said he had been lead by a remark of Kaposi to attribute much weight to certain symptoms in the diagnosis of these affections ; *e. g.*, that lupus is not so apt as syphilis to attack the bones.

DR. WIGGLESWORTH said that lupus does attack the bones, although, to be sure, not often.

DR. ATKINSON, continuing, said it was often difficult to check the ulcer of the uvula before perforation should have taken place. He considered constitutional treatment the most important in these cases. He had found most trouble in the effort to cure old recurring cases.

The PRESIDENT, DR. HYDE, of Chicago, in closing the discussion, added his testimony to that already given on the great importance of cleanliness. It was a *sine quâ non* in every case, whatever form of treatment might be adopted.

### THIRD DAY.—MORNING SESSION.

The report of the Committee on Statistics was read as follows :

### REPORT OF THE COMMITTEE ON STATISTICS FOR THE YEAR ENDING JUNE 30, 1881.

TABLE I.

| | | | | |
|---|---|---|---|---|
| District 1.—Boston Cases | Dispensary | 2,558 | Total | 3,264 |
| | Private | 706 | | |
| District 2.—New York Cases | Dispensary | 3,295 | Total | 3,837 |
| | Private | 542 | | |
| District 4.—Philadelphia Cases | Dispensary | 1,010 | Total | 1,108 |
| | Private | 98 | | |
| District 4.—Baltimore Cases | Dispensary | 503 | Total | 503 |
| | Private | 0 | | |
| District 5.—St. Louis Cases | Dispensary | 0 | Total | 264 |
| | Private | 264 | | |
| District 6.—Chicago Cases | Dispensary | 1,069 | Total | 2,100 |
| | Private | 1,031 | | |
| | Total Dispensary Cases | 8,435 | | |
| | Total Private Cases | 2,641 | | |
| | | 11,076 | | |

TABLE II.

*Showing the Returns from the respective Districts.*

| | Private Cases. | Dispensary Cases. | Total Cases. | Boston. | New York. | Philadelphia. | Baltimore. | St. Louis. | Chicago. |
|---|---|---|---|---|---|---|---|---|---|
| **Class I. Disorders of the Glands.** | | | | | | | | | |
| 1. *Of the Sweat Glands.* | | | | | | | | | |
| Hyperidrosis | 4 | 14 | 18 | | 11 | 3 | 1 | 1 | 2 |
| Miliaria crystallina | 9 | 11 | 20 | | | 6 | | 3 | 11 |
| Anidrosis | 1 | | 1 | | | | | | 1 |
| Bromidrosis | 2 | 3 | 5 | | | | | | 5 |
| Chromidrosis | | | | | | | | | |
| 2. *Of the Sebaceous Glands.* | | | | | | | | | |
| Seborrhœa: *a.* oleosa; *b.* sicca | 82 | 165 | 247 | 83 | 56 | 30 | 17 | 6 | 55 |
| Comedo | 34 | 90 | 124 | 24 | 59 | 9 | | 4 | 28 |
| Cyst: *a.* Milium; *b.* Wen | 23 | 18 | 41 | 7 | 12 | 8 | | 3 | 11 |
| Molluscum sebaceum | 10 | 10 | 20 | 5 | 5 | | | 1 | 9 |
| Diminished secretion | | | | | | | | | |
| **Class II. Inflammations.** | | | | | | | | | |
| Exanthemata | 107 | 122 | 229 | 16 | 28 | 3 | 6 | | 176 |
| Erythema simplex | 29 | 58 | 87 | 10 | 31 | 8 | 5 | 2 | 31 |
| Erythema multiforme: *a.* papulosum; *b.* bullosum; *c.* nodosum | 28 | 96 | 124 | 40 | 40 | 26 | 5 | | 13 |
| Urticaria | 42 | 302 | 344 | 75 | 174 | 54 | 8 | | 33 |
| * Dermatitis: *a.* traumatica; *b.* venenata; *c.* calorica | 57 | 183 | 240 | 42 | 109 | 36 | 15 | 4 | 34 |
| Erysipelas | 32 | 103 | 135 | 33 | 56 | 6 | 3 | | 37 |
| Furuncle | 48 | 183 | 231 | 50 | 106 | 25 | 12 | 6 | 32 |
| Anthrax | 8 | 1 | 9 | | 3 | | | | 6 |
| Phlegmona diffusa | 6 | 12 | 18 | 6 | 3 | | 3 | | 6 |
| Pustula maligna | | | | | | | | | |
| Herpes: *a.* facialis; *b.* progenitalis | 69 | 112 | 181 | 40 | 58 | 11 | 4 | 2 | 66 |
| Herpes zoster | 21 | 127 | 148 | 48 | 45 | 17 | 8 | 2 | 28 |
| Psoriasis | 92 | 250 | 342 | 83 | 128 | 47 | 11 | 4 | 69 |
| Pityriasis rubra | | 1 | 1 | | | | | | 1 |
| Lichen: *a.* planus; *b.* ruber | 5 | 21 | 26 | 1 | 12 | 2 | 3 | | 8 |
| Eczema: *a.* erythematosum; *b.* papulosum; *c.* vesiculosum; *d.* madidans; *e.* pustulosum; *f.* rubrum; *g.* squamosum | 623 | 2880 | 3503 | 1231 | 1226 | 370 | 201 | 77 | 398 |
| Prurigo | | 1 | 1 | | 1 | | | | |
| Acne | 226 | 430 | 656 | 270 | 170 | 69 | 22 | 26 | 99 |
| Impetigo | 19 | 146 | 165 | 67 | 37 | 25 | | | 36 |
| Impetigo contagiosa | 8 | 52 | 60 | 12 | 1 | 23 | 6 | 1 | 17 |
| Impetigo herpetiformis | | | | | | | | | |
| Ecthyma | 5 | 65 | 70 | 33 | 24 | 5 | 1 | | 7 |
| Pemphigus | 5 | 16 | 21 | 3 | 6 | 3 | 1 | | 8 |
| **Class III. Hæmorrhages.** | | | | | | | | | |
| Purpura: *a.* simplex; *b.* hæmorrhagica | 11 | 43 | 54 | 8 | 24 | 7 | 2 | 1 | 12 |
| **Class IV. Hypertrophies.** | | | | | | | | | |
| 1. *Of Pigment.* | | | | | | | | | |
| Lentigo | 3 | 4 | 7 | 4 | | | | 3 | |
| Chloasma: *a.* locale; *b.* universale | 32 | 28 | 60 | 18 | 22 | | 3 | 1 | 13 |
| 2. *Of Epidermal and Papillary Layers.* | | | | | | | | | |
| Keratosis: *a.* pilaris; *b.* senilis | 15 | 8 | 23 | 10 | 2 | 2 | | 3 | 6 |
| Callositas | | | | | | | | | |
| Clavus | | 6 | 6 | 1 | 2 | 1 | 2 | | |
| Cornu cutaneum | | | | | | | | | |
| Verruca | 41 | 67 | 108 | 37 | 26 | 8 | 5 | 4 | 28 |
| Verruca necrogenica | | | | | | | | | |
| Xerosis | 3 | 6 | 9 | 8 | | | | 1 | |
| Ichthyosis | 7 | 14 | 21 | 2 | 6 | 3 | 2 | | 8 |
| Of Nail | 6 | 3 | 9 | 1 | 2 | 2 | | 3 | 1 |
| Hirsuties | 30 | 2 | 32 | 12 | 3 | | | 15 | 2 |
| 3. *Of Connective Tissue.* | | | | | | | | | |
| Scleroderma | 2 | 3 | 5 | 2 | 2 | | | | 1 |
| Sclerema neonatorum | | | | | | | | | |

* Indicating affections of this class not properly included under other titles.

*Table showing the Returns from the respective Districts.*

| | Private Cases. | Dispensary Cases. | Total. | B ston. | New York. | Philadelphia. | Baltimore | St. Louis. | Chicago. |
|---|---|---|---|---|---|---|---|---|---|
| Morphœa | 5 | | 5 | | 3 | | | 1 | 1 |
| Elephantiasis Arabum | 5 | 5 | 4 | 1 | | | | | |
| Rosacea: *a.* erythematosa; *b.* hypertrophica. | 82 | 77 | 159 | 22 | 74 | 19 | 2 | 14 | 28 |
| Frambœsia | | | | | | | | | |
| **Class V. Atrophies.** | | | | | | | | | |
| 1. *Of Pigment.* | | | | | | | | | |
| Leucoderma | 4 | 6 | 10 | | 6 | | | 1 | 3 |
| Albinismus | | 2 | 2 | | | | 2 | | |
| Vitiligo | 7 | 13 | 20 | 6 | 1 | 6 | 3 | | 4 |
| Canities | 2 | 1 | 3 | 2 | 1 | | | | |
| 2. *Of Hair.* | | | | | | | | | |
| Alopecia | 68 | 22 | 90 | 62 | 14 | 4 | 2 | 3 | 5 |
| Alopecia areata | 34 | 31 | 65 | 29 | 19 | 4 | 1 | 1 | 11 |
| Alopecia furfuracea | 47 | 15 | 62 | 44 | 4 | 3 | 4 | | 7 |
| Atrophia pilorum propria | | | | | | | | | |
| 3. *Of Nail.* | | 3 | 3 | 1 | 1 | 1 | | | |
| 4. *Of Cutis.* | | | | | | | | | |
| Atrophia senilis | | | | | | | | | |
| Atrophia maculosa et striata | 2 | | 2 | | | | | 1 | 1 |
| **Class VI. New Growths.** | | | | | | | | | |
| 1. *Of Connective Tissue.* | | | | | | | | | |
| Keloid | 4 | 11 | 15 | 3 | 4 | 2 | 4 | 1 | 1 |
| Cicatrix | 2 | 8 | 10 | 2 | 4 | | 1 | 1 | 2 |
| Fibroma | | 1 | 1 | | | | | | 1 |
| Neuroma | | | | | | | | | |
| Xanthoma | 3 | 2 | 5 | 1 | 4 | | | | |
| 2. *Of Vessels.* | | | | | | | | | |
| Angioma | 21 | 26 | 47 | 16 | 7 | 3 | 4 | 4 | 13 |
| Angioma pigmentosum et atrophicum | 1 | | 1 | | 1 | | | | |
| Angioma cavernosum | | 1 | 1 | 1 | | | | | |
| Lymphangioma | 1 | 1 | 2 | | 1 | 1 | | | |
| 3. *Of Granulation Tissue.* | | | | | | | | | |
| Rhino-scleroma | | | | | | | | | |
| Lupus erythematosus | 12 | 17 | 29 | 7 | 10 | 2 | | 1 | 9 |
| Lupus vulgaris | 15 | 36 | 51 | 8 | 13 | 8 | 2 | 6 | 14 |
| Scrofuloderma | 7 | 35 | 42 | 11 | 7 | 4 | 4 | | 16 |
| Syphiloderma: *a.* erythematosum; *b.* papulosum; *c.* pustulosum; *d.* tuberculosum; *e.* gummatosum | 283 | 982 | 1265 | 273 | 454 | 85 | 23 | 16 | 414 |
| Lepra: *a.* tuberosa; *b.* maculosa; *c.* anæsthetica | 5 | 2 | 7 | 1 | 1 | | 1 | | 4 |
| Carcinoma | 36 | 51 | 87 | 21 | 27 | 10 | 6 | 5 | 18 |
| Sarcoma | | 1 | 1 | | 1 | | | | |
| **Class VII. Ulcers** | 48 | 385 | 433 | 97 | 202 | 19 | 24 | 5 | 86 |
| **Class VIII. Neuroses.** | | | | | | | | | |
| Hyperæsthesia: *a.* Pruritus; *b.* Dermatalgia. | 72 | 202 | 274 | 75 | 82 | 37 | 25 | 16 | 39 |
| Anæsthesia | 2 | 2 | 4 | | | | 1 | 1 | 2 |
| **Class IX. Parasitic Affections.** | | | | | | | | | |
| 1. *Vegetable.* | | | | | | | | | |
| Tinea favosa | 2 | 23 | 25 | 4 | 13 | 2 | 2 | | 4 |
| Tinea trichophytina: *a.* circinata; *b.* tonsurans; *c.* sycosis | 91 | 258 | 349 | 84 | 115 | 22 | 20 | 11 | 97 |
| Tinea versicolor | 26 | 98 | 124 | 23 | 44 | 15 | 8 | 3 | 31 |
| 2. *Animal.* | | | | | | | | | |
| Scabies | 7 | 127 | 134 | 22 | 64 | 11 | 6 | | 31 |
| Pediculosis capillitii | 3 | 213 | 216 | 110 | 51 | 24 | 7 | | 24 |
| Pediculosis corporis | 3 | 218 | 221 | 51 | 118 | 10 | | | 42 |
| Pediculosis pubis | 11 | 34 | 46 | 11 | 12 | 4 | | | 19 |

**Special Report on Leprosy.**

*New Brunswick.*—Dr. Graham, of Toronto, presented a brief history of the progress of leprosy in New Brunswick, drawn from the annual reports to the legislature of that Province for the last thirty years, with a valuable abstract of the report of a medical commission made in the year 1848 by Drs. Bayard and Wilson.

*California.*—Dr. Hyde presented the following account of the disease upon the Pacific Coast:

TWENTY-SIXTH ST. HOSPITAL,
SAN FRANCISCO, CAL., JULY 10, 1881.

JAMES NEVINS HYDE, M.D.,
*President Am. Derm. Association.*

MY DEAR DOCTOR:—Your favor of last month found me busily engaged on the annual report, a fact for which I would have you credit me, as against my neglect in allowing your communication to remain so long unanswered. In regard to the subject of your inquiry, leprosy, I shall be but too happy to furnish you with all the data attainable from our records, at the same time would remind you of the fact that the subjects of the disease, with one exception, have been Mongolians, and unable to give other than the most meagre account of themselves, and we are often compelled to rely upon conjecture in fixing the age of the patient.

I find by reference to the records that the first leper, *Hoy Tong*, was admitted July the 5th, 1871, and that he died on 29th of Sept., 1875.

Since that date fifty-one additional cases have been received—48 males, 3 females—making a total of fifty-two cases to account for. The hospital was designed for the treatment of small-pox exclusively, and the leprosic have been admitted only when found necessary to relieve other public institutions, or perhaps the streets of their presence.

*How Disposed of.*—Three invoices, aggregating forty-five (45), have been sent by the authorities to China. Three (3) have died of the disease. One (1) from accidental causes. One (1) escaped. One (1) committed suicide, and one (1), a Caucasian, is still an inmate.

*Ages of the Patients.*

| | |
|---|---|
| Between 15 and 20 | 4 |
| Between 20 and 25 | 9 |
| Between 25 and 30 | 17 |
| Between 30 and 40 | 13 |
| Between 40 and 50 | 9 |

No record has been kept of the duration of the disease, at the date of admission, nor of the length of time the patient had been in the country—omissions much to be regretted, and doubtless due to the fact that their detention here was but temporary, and pending shipment. It is not assumption to say they were to appearances healthy when they entered the port, the Chinese Six Companies being too keenly awake to their own interest to import unproductive labor, but that they were the subjects of the disease in its incubative stage at the date of shipment is an inference irresistable.

*Type of Disease.*

| | |
|---|---|
| Anesthetic | 14 |
| Tubercular | 9 |
| Mixed | 5 |
| Not Given | 24 |

Accompanying these notes you will find a photographic group of lepers, fourteen in number. They comprise the second shipment made to China, June the 2d, 1880. You will also find two cabinet scenes from the same group—fine specimens of the tubercular type of the disease just prior to the commencement of the ulcerative process.

*Duration of the Disease.*—From personal observation I can give no information on this point, having seen but one fatal case. In March, 1876, when I joined the hospital, it contained a young Chinaman then convalescent from varioloid. His face was covered with small tubercles (non-ulcerative), usually seen in the convalescent stage of that disease, but they did not become absorbed. He was discharged in April, but not till the similarity of these tubercles to early leprosy had been the subject of comment. On the 28th of July, 1879, he was re-admitted with tuberculous leprosy fully developed, and he died of phthisis on the 20th of the following March, four years from the first appearance of the disease. For the two years next succeeding his discharge from the hospital in 1876, he had been laboriously employed as a miner.

Per contra *Fo Lim* and *To Gan* were both inmates of the hospital two years or more; were both the subjects of the disease several years prior to admission, and were in better physical condition when discharged than when admitted. The former had been eight years in the country and four or five years the subject of the disease. Ulceration had commenced in the fossa between the lower lip and prominence of the chin, but under a more generous diet than he had been accustomed to, it soon healed, and his general health continued good during the period of his stay in the hospital.

For the past two years alimentation has been our only treatment. The leper requires good nutrition and wants it often. The ration of the American army is insufficient for his support until the last stage of the disease has been reached.

*General Prevalence.*—Any opinion as to the number of these unfortunates now in the city would be mere conjecture. I have seen none out of the Chinese quarter, and without police aid it would be useless to look for it there. It is less general than is commonly believed, yet the fact is a significant one, that, on the 2d of June, every known leper in the city was shipped for China, and before the end of the year fourteen new cases have accumulated upon our hands. The Chinese Consul claims hospital rights for these people; the authorities resist the claim, and the courts are now adjudicating upon the question.

*Is it Contagious?*—English writers in India say it is not, or at least not in the sense in which the term is usually employed; but the Chinaman who has been familiar with the disease and its traditions for centuries, avoids the leper with great care, and will stand for hours rather than occupy a seat that has been vacated by one.

*Heredity.*—The leper maintains the most profound silence upon this subject. I have seen but one who admitted its existence as a family taint, and he would, it is probable, have denied it, had not the inquiry been made prior to a conference with his fellows in the lazarette.

*Incubation.*—*Ah Yong* had been five years, *Ah Tong* three years, and *Lee Fong* (in whom the disease had but recently declared itself) five years in the country before any symptom appeared. *Tu Wuog*, for several years a marine fireman in the employ of Pacific Mail Co., between this port and the Orient, a man of fine physique, and well nourished, developed the disease at 32, after five years of generous diet on board an American steamship, and surrounded by healthful sanitary conditions. *Thos. Stanton*, present age 47, after a residence of nine years in Madras, Ceylon and Bombay, sailed from Calcutta for New York, December 24, 1870. For three and a half years after his arrival he remained in good health and worked as stone-mason's assistant in Cleveland, Nashville, and other western cities. In the autumn of 1874, becoming the subject of rheumatic pains, he concluded to seek a milder climate, and going to Louisiana he remained there and in other of the cotton States till the spring of 1878. During his stay in the South he had worked a portion of the time, " but did not feel well at all ;" and, although he had no medical advice, he " knew it must be that his blood was out of order, or these pimples wouldn't have come out on his face" (leprosic tubercles). Concluding to work his way back to Bombay, he reached Sacramento during the vintage of '78, and obtained employment in the drying-room of a raisin factory. He was the only white man in the room. During the second day his fellow-workmen discontinued their work and declined to resume until the foreman was discharged. The Chinamen had recognized the disease—more successful in this particular than one of the visiting surgeons of a hospital, who, for a period of six months subsequent, treated him as a syphilitic. He was admitted to this hospital March 4, 1880, with fully developed leprosy of the mixed variety.

Six months since his appetite failed him. The numbness in the toes, of which he had formerly complained, gradually extended up the feet ; his voice became husky, cough troublesome ; the muscles are wasting, the toes sloughing ; he has become very irritable, and he will, it is probable, succumb to the disease during the current year. In this instance, the only one in which I have been able to obtain a detailed history, the earliest symptom of the disease made its appearance four years after leaving the locality where it is assumed to have been acquired.

*Symptoms.*—No better description need be looked for than that given by Dr. Tilbury Fox, on pages 312 and 313 of his able work on the skin. Of the functional lesion in the parts supplied by the ulnar nerve, I will state that in the group of fourteen I send you it was strongly marked in nine. and a very good idea of the changes which followed can be obtained by examining them (slightly magnified) in the two arm views.

Before closing, I desire to express my regret that I am not able to procure for you photographs of the cabinet size, but I find they are out of print and the negatives lost or rendered useless.

I am, my dear Doctor, respectfully your obd't servant,

JOHN W. FOYE, M.D.

In *Oregon*, also, the disease has appeared among the Chinese immigrants, steps having been taken in February last by the authorities to remove five lepers from the Portland poor-farm and to re-ship them to China.

DR. CHARLES HEITZMANN, of New York, read a paper entitled

### Clinical Experience in the Use of the Solution of Oxysulphuret of Calcium.

This solution, commonly called after the name of Dr. Vlemingckx, who first introduced its use, has been long known in dermatology. It has been used chiefly for the treatment of scabies, and by Hebra for the removal of patches of psoriasis.

The solution of oxysulphuret of calcium is prepared as follows: Take one part of quicklime, two parts of precipitated sulphur, and twenty parts of water ; boil in a china or glass vessel to the remnant of twelve parts, and filter. The dark-brown liquid resultant has a disagreeable smell and is a strong caustic. Oil of anise will in part obviate the objectionable smell. The solution may be diluted with water or alcohol, the latter precipitating the sulphur, but not materially altering its composition.

The writer had used it in thirty cases of psoriasis of the body, the solution being applied in full strength or diluted, according to the susceptibility of the patient's skin, being rubbed into the patches with a piece of flannel until slight stinging ensues. Care must be taken not to irritate the skin by a too strong solution. Tar ointment is afterward to be rubbed into the patches.

The writer had employed the solution of oxysulphuret of calcium in ninety-five cases of acne disseminata, of which seventy-five were cured ; in twelve the result was unknown, and in three cases the disease was aggravated. The solution is in these cases to be diluted with three to six parts of water, rubbed into the face with a flannel rag, and left on over night.

The next morning the face is washed with soap and water. The solution may be employed of greater strength as the patient becomes accustomed to it. Cold-cream may be used to disguise the slight desquamation.

The writer had also employed the solution in a few cases of chronic eczema, fourteen in all. In four cases of chronic eczema of the scrotum, the application of dilute Vlemingckx's solution, gradually strengthened, resulted in a cure.

Thirty-six cases of rosacea were treated by the writer, with good result in twenty-nine cases.

A limited number of cases of tinea tonsurans were treated by means of the solution, but without beneficial result. Eleven cases of tinea versicolor were treated, with brilliant success in every instance, the solution being used in half strength. It appears to be the safest and surest remedy in this affection.

Finally, six cases of scabies were treated, all successfully.

DR. F. B. GREENOUGH, of Boston, asked the writer if the solution was to be applied over the whole face in acne, or to the lesions only. Dr. Heitzmann said it was to be applied over the whole face.

DR. WHITE, of Boston, said he had formerly used Vlemingckx's solution a great deal, but of late years had used it but little. Patients object to the remedy, and it is difficult to get them to use it long enough to do good. He was at present accustomed to use other preparations of sulphur which are less objectionable. Vlemingckx's solution is apt to produce excessive action when used strong enough to be beneficial. He had never used it in acne, and thought it doubtful if he ever should. His patients, mostly young ladies would, he was quite sure, object to the employment of so disagreeable a remedy, and moreover metallic articles in the neighborhood are apt to be affected by the fumes, which makes it very objectionable. He would, however, agree with Dr. Heitzmann as to its value in acne. As regarded the use of sulphur preparations in the treatment of pigment anomalies (hypertrophy), Dr. White did not consider them efficacious ; much less so, certainly, than other remedies.

DR. HEITZMANN said, in reply to Dr. White, that thin-skinned persons did not, in his experience, object to the use of Vlemingckx's solution, and inquired what alkaline sulphur remedy Dr. White used.

DR. WHITE said that he sometimes used sulphur and sapo viridis.

DR. HEITZMANN said he had tried sulphuret of potassium, but had not obtained good results.

DR. WIGGLESWORTH, of Boston, said that the mechanical, as well as the medicinal, treatment of skin diseases should always be kept in mind as of importance. He expressed his preference for sulphur ointments as bland and soothing. He was also accustomed to use on occasions carbonate of potassium and sulphur.

DR. DUHRING, of Philadelphia, inquired of Dr. Heitzmann regarding the amount of scaling in cases of acne treated by Vlemingckx's solution, and asked if he followed it up with bland ointments.

DR. HEITZMANN replied that the amount of scaling depended very much on the character of the given case. He always began with a diluted solution, gradually increasing its strength.

DR. HEITZMANN then read a paper entitled

### Remarks on Akido-galvano-cautery (Electrolysis) for Epilation.

Referring to a paper by himself, giving experiments in the injection of caustic liquids for the removal of superfluous hairs, and read at a previous meeting of the Association, the writer said that these experiments had resulted unsatisfactorily in the case reported. The lady had since then consulted prominent Viennese dermatologists, who assured her that no cure could be effected in her case, and that any one who should promise such a cure would be a charlatan.

The discussion on that paper by members of the Association present at the time, had re-drawn the writer's attention to the method termed by its inventors, Drs. Hardaway and Michel, of St. Louis, "Electrolysis," and he had employed it in the case referred to. The term "electrolysis" is objectionable, as it involves the idea of an electric action upon the tissues without thermic influence. The existence of such an action has, however, as yet not been proved. What we use for the destruction of the hair bulb is, in reality, the galvano-cautery. The action being induced by a delicate needle, the writer suggested prefixing the word "Akis," and designating the method "Akido-galvano-cautery," or "Akido-cautery." The credit of the invention of the method belongs to Dr. Michel, of St. Louis, and it was first applied to the destruction of the hairs of the face by Dr. Hardaway, of St. Louis (in a paper read before this Association in 1878). Dr. George Fox deserves credit for certain improvements in the method.

Dr. Heitzmann, the writer, uses the "chloride of silver" battery of the "Electro-medical Company," of Union Square, New York, consisting of twenty cells in a perfectly closed case, neat-looking, easily transportable, and needing no filling for one to two years. He finds five or six cells sufficient, and the operation successful and almost painless ; several minutes, however, being required to destroy each hair bulb. The negative pole, armed with a dentist's brooch, is applied to the bottom of the hair pouch, the positive pole, armed with a wet sponge, being held in the patient's hand. Very soon after the current is closed, a whitish discoloration is noticed around the needle, then flakes of epidermis become detached from the mouth of the hair pouch, a little frothing appears, with marked redness about the needle Repeated gentle trials with the tweezers finally succeed in detaching the hair without the least force, the root of the extracted hair looking as if burned through, and slightly discolored. The hair pouch is gaping, and a second insertion of the needle into the pouch for a few moments is an additional security for the complete eradication of stronger hairs. A wheal appears immediately after the operation, and a small papula or pustule on the succeeding days, which soon disappears without leaving a noteworthy disfiguration.

Some hairs return again. These are usually sufficiently large within three weeks to justify a second operation.

During the winter months of 1880-81 Dr. Heitzmann removed 450 hairs from the patient operated upon, leaving only downy hairs behind. Three months later only six of these had again reappeared. The result of this new American method is brilliant indeed, and honor is due to our brethren of the Dermatological Association who have initiated and perfected the procedure.

DR. WHITE, of Boston, questioned the accuracy of Dr. Heitzmann's statement that the influence of the cautery is purely thermal. He thought it electrolytic. He (Dr. White) had operated by the method described in ten cases during the past year. He had not succeeded so well in his earlier cases as in the more recent ones. He was accustomed to employ a stronger current than Dr. Heitzmann had used—fifteen cells. He usually placed the positive pole as near as possible to the needle, and kept it under his own control. With all precautions, many cases must be gone over again after three weeks. Dr. White said that, in his experience, scars are usually left ; he thought that usually essential to the cure. In some cases, however, he did not find a scar.

DR. DUHRING, of Philadelphia, said, in reference to Dr. White's opinion with regard to the action of the cautery, that he thought it both thermic and electrolytic. He had seen the needle brought to a red heat. He had, after a prolonged trial, abandoned the use of the dental nerve brooch, and now used a fine cambric needle, ground down by a skillful workman. He had devised a light and convenient instrument, made by Fleming, of Philadelphia, for holding the needle, and which he had described and figured in the July number of the *American Journal of the Medical Sciences.* He thought that the operation demanded peculiar manual skill, and considered himself as succeeding at present much better than formerly. Sometimes he found scarring to result, and sometimes not.

DR. WHITE said he never employed thermal influence. His needle was always cold to the touch. He had had no trouble with the dental brooch, which was flexible and elastic. He asked if Dr. Duhring's brooches had been of foreign manufacture.

DR. DUHRING said they had been of foreign make. He inquired if Dr. White did not find pain caused by applying the positive and negative poles so near each other.

DR. WHITE said that he had not found pain result from the procedure mentioned. He had used the chloride of silver battery employed by Dr. Heitzmann, and was well pleased with its performance. He inquired if any of the members had observed the growth of new hairs as a result of the application of the positive pole to the skin.

None of the members had observed this phenomenon.

### REPORT OF THE COMMITTEE ON NOMENCLATURE AND CLASSIFICATION

The Committee on Nomenclature and Classification reported that no changes were suggested. On motion the report was accepted.

THE REPORT OF THE SPECIAL COMMITTEE ON MICROSCOPIC EXAMINATION OF A SPECIMEN OF AINHUM

was then read by the Chairman, DR. HEITZMANN, of New York, as follows :

The specimen submitted to your committee consisted of the small toe of a negro from Brazil, which by constriction on the level of the fold between the toe and the foot, had been separated from the body. It represents an ovoid mass, about two inches in longitudinal, and one and a half inches in transverse diameter, of a dark brown color, the surface being nodulated, and especially on the distal end supplied with distinct rows of papillæ. On the dorsal surface of the distal end there is a well-preserved nail; in the midst of the plantar surface there is a roundish field, one quarter inch in diameter, uncolored and uneven, the place where the pedicle was located.

Frontal and sagittal sections made through this mass, to the naked eyed exhibit a very broad epithelial investment all around, except on the place of the former pedicle. The epithelial investment consists of an outer, dark brown, and an inner, colorless layer, the boundary line between these two portions being highly scalloped. The papillary layer is enlarged to the diameter of two millimeters, and the papillæ are so much elongated as to be plainly perceptible to the naked eye.

The main bulk of the toe is composed of coarse bundles of connective tissue in a reticular arrangement, and the meshes inclosed by the bundles hold some fat. The layer next to the bone, evidently the thickened periosteum, is produced by a very dense fibrous tissue, without reticular structure. On the frontal section the phalanges, of which only the second and the third are imbedded in the mass of the toe, look entirely displaced, so as to assume a direction outward and upward, in an acute angle of nearly 45°. The diameters of the phalanges are not noticably changed. On the outer surface of the phalanges there is a broad layer of tendinous tissue attached to the periosteum. This tendon occupies the central portion of the place of detachment, where it terminates in a rounded-off extremity, a little over one millimeter in width. Around the end of the tendon both the derma and the epithelial layers terminate with sloping ends, which slightly protrude around the tendon.

Sections made in a vertical direction with the razor, under the microscope exhibited a considerable widening of the epithelial layers, which consist of several strata of alternating horny and protoplasmic epithelia. In the horny portion there are scattered fat-globules in moderate quantity. The papillæ are very much elongated, though not noticeably widened in their transverse diameter except on their bases. Horizontal sections give beautiful figures of the transversly-cut papillæ, and the very broad, concentrically arranged layers of the epithelia.

The derma, which, as seen by the naked eye, is the formation largely prevailing, consists of coarse bundles of fibrous connective tissue, in a very irregular arrangement. The meshes left between the bundles hold either fat-globules or a myxomatous connective tissue, crowded with medullary or inflammatory corpuscles. Such corpuscles are visible throughout the fibrous portion also, but here in a much smaller number. Scanty remnants of the coils of the sudoriparous glands are visible, and also ducts of such glands. The arteries traversing the derma are surrounded by a very distinct, as a rule, broadened layer of smooth muscles, while their calibre, on an average, is narrowed. The veins, on the contrary, are without exception considerably enlarged and completely filled with blood. The periosteum ensheathing the phalanges consists of a dense fibrous connective tissue, whose bundles are rectangularly interlaced and hold a relatively small number of inflammatory corpuscles. The portion of the periosteum nearest to the bone is to a great extent myxomatous in nature, and in some places crowded with inflammatory corpuscles.

The surface of the bone, and also the trabeculæ of the cancellous portion, are irregularly jagged, supplied with lesions and bay-like excavations, which either are filled with a fully developed connective tissue, or with masses of inflammatory corpuscles. The picture reminds of the cementum of deciduous teeth, whose surface, at the time when the tooth becomes reduced, melted down, looks in a very similar way. The medullary spaces of the cancellous bony tissue are filled either with fat-globules, or with a myxomatous connective tissue, the veins being also dilated and engorged with blood-corpuscles.

The articular cartilage on the lower extremity of the second phalanx is considerably reduced in size. Its surface on many places looks crowded like that of the bone, and the bay-like excavations are filled either with inflammatory corpuscles or a fully developed connective tissue.

Lastly, it is worth mentioning, that on some places in the fat-tissue the fat-globules are separated from each other by large spaces, filled with a finely granular mass, namely, coagulated albumen. This image corresponds to that of œdema.

The result of the researches of your committee is, that the specimen of ainhum fully answers the process that we are accustomed to call hypertrophy or hyperplasia of the tissues due to a chronic inflammatory process. The hyperplasia involves both the epithelial investment of the skin and the derma, while the bony and cartilaginous tissue, due to the slow inflammation, is gradually reduced into fibrous connective tissue. As to the cause of this marked hyperplasia of the toe, your committee submit the following suggestions :

The description of the process by Drs. Da Silva Lima and J. Nevins Hyde, draws attention to a hard constricting ring on the plantar surface of the foot in the fold, which demarcates the toe from the sole of the foot. This fold strictly corresponds to the articulation of the first with the second phalanx of the toe, and a gradual constriction of the tissues necessarily will lead to a separation in the named articulation. To what is this constriction due? Is it an inflammatory process in the derma, or is it an atrophic process in the derma, kindred to that of lepra mutilans? Nothing of the kind is indicated by the specimen. The derma noticeably slopes in the immediate vicinity of the strangulated place, whose middle portion, as mentioned above, is occupied by the flexor tendon ; there are no signs of an inflammatory thickening on the place of the constriction.

Furthermore, how could a primary constriction of the derma explain the remarkable deviation of the two phalanges within the stump? It was said before that the phalanges stand in an acute angle outward and upward. Can a narrow constricting ring ever produce such a deviation of bony formations?

The probability, in fact, is that the constricting ring is produced artificially, by a thin ligature tied around the toe, if not continually, at least for certain periods. The deviation of the phalanges, and the changes within the tissues, indicate a self-mutilation rather, done with a purpose and persistence, than a disease of a specific nature.

C. HEITZMANN, I. E. ATKINSON, *Members of the Committee.*

The report of the committee was on motion accepted.

**Exhibition of Specimens.**

Various microscopic specimens, illustrating the several papers read at the meeting, were then placed under several microscopes and demonstrated to the members of the Association. After considerable discussion on these, the newly-elected officers of the Association for the year 1881–2 were duly installed, and the Association then adjourned, to meet in Newport, R. I., on September 1, 2 and 3, 1882.

---

NERVOUSNESS RESULTING FROM INTEMPERANCE.—We have found Celerina exceedingly valuable in the treatment of nervous headache, nervous exhaustion, and other associated ailments of women : but the cases to which we now desire to call attention, where the Celerina is of inestimable value, are those suffering from nervousness resulting from intemperance. Every practitioner meets with such cases. Men, and sometimes women, come to us trembling and apparently exhausted, all from the effects of intemperance. Such cases are approaching delirium tremens. Celerina is the most appropriate prescription we can give them. A few doses of bromide of potassium may be given, alternated with the Celerina, at first; but after this, for permanent effects, we depend upon the Celerina.—*American Med. Journal.*

MAYOR LATROBE, of Baltimore, has signed an ordinance absolutely prohibiting the sale of toy pistols in that city. The large number of deaths by lockjaw last summer prompted the passing of the act.—*Boston Med. and Surg. Journal.*

RESECTION OF SMALL INTESTINE.—Kœberli has reported to the French Academy cases of resection of the small intestine, and advocates this operation. This is not new; the operation was successfully performed at Manassas Junction, Va., during the war.—*Medical News.*

